# Unmet weight loss targets in real-world clinical practice: weight management and perceptions in China

**DOI:** 10.3389/fendo.2024.1470394

**Published:** 2024-11-07

**Authors:** Ziwei Lin, Si Si, Jia Liu, Hao Zhu, Jiawei Xu, Esther Artime, Swarna Khare, Victoria Higgins, Andrea Leith, Shen Qu

**Affiliations:** ^1^ Eli Lilly and Company, Suzhou, China; ^2^ Endocrinology and Metabolism Center, Shanghai Tenth People’s Hospital, School of Medicine, Tongji University, Shanghai, China; ^3^ Eli Lilly and Company, Alcobendas, Spain; ^4^ Eli Lilly and Company, Bracknell, United Kingdom; ^5^ Adelphi Real World, Bollington, United Kingdom; ^6^ Endocrinology Metabolic and Thyroid Center, SinoUnited Health, Shanghai, China

**Keywords:** China, dietary intervention, obesity therapy, real-world evidence, weight control

## Abstract

**Aims:**

To describe weight management and perceptions in China.

**Materials and methods:**

Data were from the Adelphi Real World Obesity Disease Specific Programme™, a cross-sectional survey between April and July 2022 of physicians managing people with obesity or overweight (PwO) and PwO in real-world clinical practice in China. At data collection, eligible PwO were aged ≥18 years, under weight management and/or had a body mass index (BMI) ≥28 kg/m^2^.

**Results:**

In total, 100 physicians and 801 PwO were enrolled. More than two thirds of PwO (70.7%; 531/751) were not diagnosed with obesity until a BMI ≥30 kg/m^2^. Most PwO (78%; 625/801) were on treatment for at least one obesity-related complication (ORC). Physicians commonly initiated weight loss discussions with PwO who already had an ORC (48.0%; 48/100). According to physicians and PwO, the mean target BMI was set at 25.8 kg/m^2^ and 24.3 kg/m^2^, and the mean target percentage weight loss was 19.6% and 23.7%, respectively. Over a median 6.4 months of weight management, the mean percentage weight loss was 4.1%. Few PwO achieved the weight loss target set by their physician (9.9%; 69/695) or themselves (2.0%; 14/696). Most physicians and PwO were unsatisfied with the current weight loss (92.3% [739/801] and 82.0% [650/793], respectively).

**Conclusions:**

These findings suggest that earlier intervention may be needed to address obesity as a disease. Most physicians and PwO recognized the importance of normal weight, but few PwO achieved weight loss targets, which may suggest an unmet need for improved weight management.

## Introduction

In China, the prevalence of obesity and overweight has increased rapidly over the past four decades, presenting a major public health issue (1). A nationwide cross-sectional study of 15.8 million Chinese adults conducted in 2019 showed that, based on Chinese criteria, 14.1% of the population had obesity (body mass index [BMI] ≥28 kg/m^2^), while a further 34.8% had overweight (BMI 24 to <28 kg/m^2^) (2). Obesity and overweight are associated with an increased risk of metabolic disease, cardiovascular disease, and some malignancies (3, 4). In China, the death rate from obesity (defined as BMI ≥30 kg/m^2^ according to international criteria) was estimated at 40.6 per 100,000 people in 2019, accounting for 6.4% of total deaths and making obesity the eighth highest risk factor for death (5).

The challenges to control the obesity problem in China are multifold. Firstly, public awareness of weight management is low. Despite the high prevalence of obesity and associated public health burden (1), people with obesity or overweight (PwO) and healthcare professionals have a poor understanding of the need for weight control to improve health conditions, rather than merely to address cosmetic issues (4, 6). Chinese media coverage tends to communicate that obesity is a lifestyle issue that is easily remedied by behavioral changes, which may increase the difficulty of obesity management (7). Moreover, in traditional Chinese culture, excess bodyweight is believed to represent health and prosperity (8, 9), and PwO may misperceive their weight status (10, 11), presenting a further barrier to weight loss attempts. Secondly, weight management in clinical practice is suboptimal. Obesity-related clinical guidelines, policies and programs have been introduced in China in response to a growing realization that obesity is a chronic relapsing disease requiring long-term management (6, 12). However, existing measures are insufficient to control the obesity problem and clinical efforts are hampered by the lack of official recognition of obesity as a disease unless accompanied by major comorbidities, especially in lower tier healthcare centers and remote regions not equipped to provide obesity care (6, 12). In China, the initial goal for PwO is 5-15% weight loss in the first 6 months (6). For PwO who achieve their weight loss goals, long-term (≥1 year) weight loss maintenance programs are recommended (13). Nonetheless, the management and treatment of obesity in Chinese clinical practice remains unclear. Lifestyle intervention programs are recommended as a first-line treatment for obesity, although there is no robust evidence of their effectiveness in Chinese populations (6). Alternative options include pharmacotherapy and bariatric surgery, but the availability of weight-loss medications in China is currently limited and acceptance of surgical interventions is low (4, 6).

The Adelphi Real World Obesity Disease Specific Programme™ (DSP™) aimed to generate real-world data from clinical practice across different countries on the patient journey and treatment landscape for PwO. The objective of this analysis was to describe obesity awareness and weight loss triggers, weight loss targets, actual weight loss, and the gap between target and actual weight loss in real-world clinical practice in China. An understanding of current weight management pathways and perceptions may help to bridge this gap and inform future clinical practice and drug development.

## Materials and methods

### Study design and data sources

Data were drawn from the Adelphi Real World Obesity DSP conducted in China between April and July 2022. DSPs are cross-sectional surveys with retrospective data capture that include physicians and their PwO presenting in real-world clinical settings and have previously been described, validated and shown to be representative and consistent over time (14–16). DSPs are designed without a prior hypothesis to holistically and impartially understand patients, the impact of their disease and associated treatment/management approaches in real-world settings, reflecting current real-world clinical practice. Briefly, data were collected from physician surveys, detailed physician-reported patient questionnaires, and patient-reported questionnaires all designed by a dedicated multi-disciplinary project team at Adelphi Real World.

Ethics exemption was obtained from Pearl Institutional Review Board (Approval No. #22-ADRW-136). All PwO provided written informed consent for use of their data, which were anonymized and aggregated. This research did not involve provision of medication, tests or investigations, or development or testing of a hypothesis.

### Study population

All participating physicians were specialists (Internal Medicine and Endocrinologists) with personal responsibility for the management and treatment of PwO, had a minimum workload of 16 PwO in a typical month, and met all survey requirements. To avoid selection bias, physicians were randomly chosen from publicly available lists of Chinese healthcare physicians. Each physician was instructed to complete a physician survey and a physician-reported patient questionnaire for the next eight PwO attending their clinic who they deemed to meet the inclusion criteria. The number of PwO was chosen to maximize the number of physicians sampled, while minimizing the burden on each physician. Eligible PwO were aged ≥18 years, currently enrolled in a weight management program and/or having a body mass index (BMI) ≥28 kg/m^2^, and were not involved in a clinical trial at the time of data collection. The same PwO for whom physicians completed physician-reported questionnaires were asked to complete patient-reported questionnaires on a voluntary basis. To ensure that responses were confidential, PwO completed questionnaires independently of their physician immediately after the consultation and returned them in a sealed envelope. No personally identifiable data were collected. Physician and patient data were pseudonymized by assigning a code to allow for matching of patient and physician responses. Of note, during the time window of this survey, no approved prescription-only anti-obesity medications (AOMs) were available in China; orlistat was the only medication approved for weight loss management and was available over the counter (OTC). However, PwO receiving off-label metformin, glucagon-like peptide-1 receptor agonists or OTC AOMs were included.

### Outcome measures

#### Physician survey

In an online survey, physicians were asked to indicate their medical specialty and recall the overall trends for obesity management, number of PwO seeking health care, and the current clinical practice for weight management based on their clinical experience.

#### Physician-reported patient questionnaire

Physicians were then required to complete a physician-reported patient questionnaire providing basic demographic and medical information based on medical charts for participating PwO, as well as their evaluations and expectations for the weight loss journeys to date. Data collected through these questionnaires had been captured by physicians during routine patient consultations.

#### Patient-reported questionnaire

The same PwO were asked to self-report their weight loss target and weight loss journey for their current weight loss attempt, as well as experiences of past weight loss attempts, including number of attempts, weight loss journey and reasons for discontinuing attempts at weight loss. Completion by the patient was voluntary and non-completion did not exempt provision of the physician-reported patient questionnaire.

### Statistical analysis

All analyzes were descriptive. Categorical variables were reported using frequencies and percentages and continuous variables were reported as mean and standard deviation (SD) or median and interquartile range (Q1, Q3). Characteristics of the physician and PwO populations, obesity awareness and triggers for initiating weight loss attempts, weight loss targets set by physicians and PwO, and the current weight loss journey for PwO were described. Physicians and PwO were asked to indicate weight loss targets at the start of the current weight loss attempt in the form of absolute value of weight, from which BMI targets and percentage weight loss targets were calculated. BMI and percentage weight loss targets were analyzed by BMI categories at the start of the current weight loss attempt (24.0–27.9 kg/m^2^, 28.0–29.9 kg/m^2^, 30.0–34.9 kg/m^2^, and ≥35.0 kg/m^2^). The anticipated speed of weight loss according to the physician was calculated as the target percentage weight loss divided by the anticipated time to achieve target weight.

## Results

### Characteristics of physician and PwO populations

In total, 100 physicians and 801 PwO in China were enrolled. Of the 801 participating PwO, 795 filled out patient-reported questionnaires. Characteristics of the participating physicians and their workloads are shown in **Table 1**. Of the 100 participating physicians, 50 specialized in endocrinology and 50 in internal medicine. The median (Q1, Q3) number of PwO consulted per physician per month was 50 (28, 83).

**Table 1:** Physician survey (N=100).

	Value
Specialties, n (%)	
Endocrinology	50 (50.0)
Internal medicine	50 (50.0)
Per recall in the past 3 years, the number of PwO, n (%)	
Increased	77 (77.0)
Remained unchanged	20 (20.0)
Decreased	3 (3.0)
Per recall in the past 3 years, the proportion of PwO seeking care, n (%)	
Increased	71 (71.0)
Remained unchanged	25 (25.0)
Decreased	4 (4.0)
Number of PwO consulted per physician per month, median (Q1, Q3)	50 (28, 83)
Number of PwO newly diagnosed with obesity per physician in the last 12 months, median (Q1, Q3)	35 (20, 80)
Categorization of PwO newly diagnosed in the last 12 months, median (Q1, Q3)	
% PwO with no significant comorbidities despite their weight	42.9 (30.0, 61.6)
% PwO with significant comorbidity resulting from/made worse by their weight	34.7 (25.0, 50.0)
% PwO with significant comorbidity which caused/contributed to their weight	16.7 (10.4, 25.0)
Most frequent reason to initiate a first discussion about weight, n (%)	
PwO already had an ORC	48 (48.0)
PwO at risk of developing an ORC	46 (46.0)
PwO expressing concern about their weight	5 (5.0)
PwO suffering with mobility issues resulting from their current weight	1 (1.0)
Agreement with the statement “It’s important to manage and treat obesity as early as possible to prevent further complications”, n (%)	
Strongly agree	66 (66.0)
Somewhat agree	23 (23.0)
Neither agree nor disagree	10 (10.0)
Somewhat disagree	1 (1.0)
Strongly disagree	0
Agreement with the statement “Obesity is brought about by poor lifestyle choices”, n (%)	
Strongly agree	33 (33.0)
Somewhat agree	48 (48.0)
Neither agree nor disagree	13 (13.0)
Somewhat disagree	6 (6.0)
Strongly disagree	0

ORC, obesity-related complication; PwO, people with obesity or overweight.

Characteristics of the PwO population are shown in **Table 2**. The mean (SD) age was 38.3 (11.7) years and 55.4% (444/801) were female. The median (Q1, Q3) reported duration of diagnosed obesity was 13.3 (5.5, 27.5) months. Most PwO (78%; 625/801) were on treatment for at least one obesity-related complication (ORC). The most common ORCs were dyslipidemia (36.7%; 294/801), dysglycemia (i.e. type 2 diabetes, pre-diabetes, or impaired glucose tolerance) (29.7%; 238/801), and hypertension (29.6%; 237/801). The mean (SD) number of comorbidities for the total patients was 1.9 (1.7).

**Table 2:** Physician-reported PwO characteristics.

Characteristics	N^†^	Value
Age, mean (SD)	801	38.3 (11.7)
Sex, n (%)	801	
Male		357 (44.6)
Female		444 (55.4)
Smoking status, n (%)	782	
Never smoked		508 (65.0)
Current smoker		188 (24.0)
Ex-smoker		86 (11.0)
Socioeconomic status,^‡^ n (%)	800	
Low		482 (60.3)
Indeterminate		69 (8.6)
High		249 (31.1)
Employment status, n (%)	801	
Working full-time		561 (70.0)
Working part-time		28 (3.5)
Homemaker		50 (6.2)
Unemployed		33 (4.2)
Retired		81 (10.1)
Student		48 (6.0)
Education level, n (%)	775	
Did not complete high school		80 (10.3)
High school		192 (24.8)
College		462 (59.6)
Master, PhD, or postdoctoral		41 (5.3)
Under health insurance coverage, n (%)	798	
Public health insurance		556 (69.7)
Private health insurance		19 (2.4)
No health insurance		223 (27.9)
Reported duration of diagnosed obesity, months, median (Q1, Q3)	755	13.3 (5.5, 27.5)
Number of diagnosed comorbidities, mean (SD)	801	1.9 (1.7)
Major ORCs being treated before current weight loss attempt (multiple choice), n (%)	801	
None		176 (22.0)
Dyslipidemia^§^		294 (36.7)
Type 2 diabetes/pre-diabetes/impaired glucose tolerance		238 (29.7)
Hypertension		237 (29.6)
Stress/tiredness/anxiety/depression		129 (16.1)
Hyperuricemia/gout		79 (9.9)
Non-alcoholic fatty liver disease/non-alcoholic steatohepatitis		69 (8.6)
Insulin resistance		62 (7.7)
Coronary heart disease		32 (4.0)
Sleep apnea		28 (3.5)
Osteoarthritis		27 (3.4)
Polycystic ovary syndrome		26 (3.2)
ORCs triggered weight loss decision in PwO with ORCs, n (%)^¶^	625	
Yes		423 (67.7)
No		164 (26.2)
Don’t know		38 (6.1)

^†^The denominator depends on the actual number of PwO for which each question was answered.

^‡^Defined according to education level (low = did not complete high school, completed high school, or 2 or 3 years of junior college; high = 4 years of college-Bachelor, Master, PhD, or postdoctoral), insurance status (low = urban, urban resident, or new rural cooperative medical insurance; high = commercial insurance), and employment status (low = unemployment; high = full- or part-time employment). These variables were imputed into an algorithm to determine a PwO’s socioeconomic status (low, indeterminate, or high) based on a ‘majority wins’ approach.

^§^High triglyceride, total cholesterol, and/or low-density lipoprotein cholesterol levels; and/or low-high density lipoprotein cholesterol levels.

^¶^Denominator is patients with at least one ORC under treatment.

ORC, obesity-related complication; PwO, people with obesity or overweight; SD, standard deviation.

### Obesity awareness and weight loss triggers

Over the past 3 years, most physicians recalled an increase in the numbers of PwO (77.0%; 77/100) and of PwO seeking health care (71.0%; 71/100) (**Table 1**). Physicians recalled that more than half of PwO actively seeking healthcare had ORCs, with a median (Q1, Q3) of 34.7% (25.0%, 50.0%) having comorbidities resulting from or made worse by their weight, and a median (Q1, Q3) of 16.7% (10.4%, 25.0%) having comorbidities that caused or contributed to their weight (**Table 1**). Accordingly, of the PwO who were on treatment for at least one ORC, 67.7% (423/625) had decided to start a weight loss program due to ORCs (**Table 2**). In theory, most physicians agreed that it was important to manage and treat obesity as early as possible to prevent further complications (89.0%; 89/100) (**Table 1**). However, in clinical practice almost half of physicians (48.0%; 48/100) waited for the onset of an ORC to initiate the first discussion about weight (**Table 1**). Most physicians (81.0%) agreed that obesity was caused by poor lifestyle choices (**Table 1**).

### Weight loss targets

**Figure 1:**
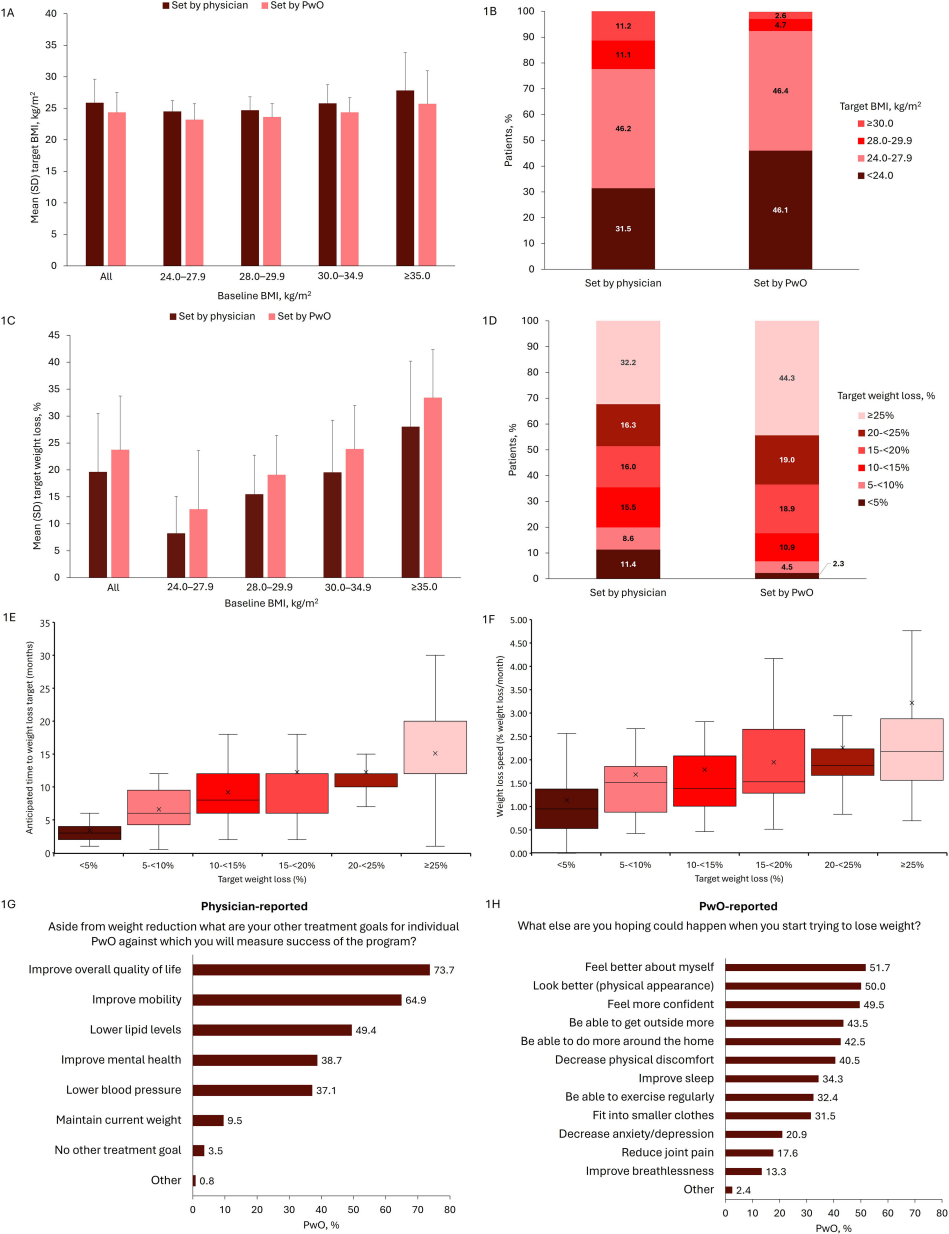
Weight loss targets set by physicians and PwO at the start of the current attempt **(A)** Target BMI by BMI category at current attempt start, **(B)** Target BMI categories, **(C)** Target percentage weight loss by BMI category at current attempt start, **(D)** Target percentage weight loss categories, **(E)** Anticipated time to achieve weight loss target, **(F)** Average speed of weight loss, **(G, H)** Other physician and PwO goals beyond weight reduction. PwO populations: **(A, C)** All: n=695 for physician-reported, n=688 for PwO; 24.0–27.9 kg/m^2^: n=43 for physician-reported, n=47 for PwO; 28.0–29.9 kg/m^2^: n=161 for physician-reported, n=172 for PwO; 30.0–34.9 kg/m^2^: n=349 for physician-reported, n=339 for PwO; ≥35 kg/m^2^: n=142 for physician-reported, n=130 for PwO. **(B, D)** n=695 for physician-reported, n=688 for PwO. **(E, F)** <5%: n=76; 5–<10%: n=56; 10–<15%: n=100; 15–<20%: n=96; 20–<25%: n=102; ≥25%: n=131. **(G)** n=801. **(H)** n=788. For **(E, F)** x represents the mean, the band inside the box represents the median, the bottom and top of the box represent the first and third quartiles, and the ends of the whiskers represent the range. BMI, body mass index; PwO, patients with obesity or overweight; SD, standard deviation.

At the start of the current weight loss attempt, most PwO had clear weight targets set by the physician (86.8%; 695/801) and by themselves (85.9%; 688/795). Weight loss targets set by physicians and PwO at the start of the current weight loss attempt are shown in **Figures 1A–D**. Across all PwO, the mean (SD) target BMI was 25.8 (3.8) kg/m^2^ according to physicians and 24.3 (3.2) kg/m^2^ according to PwO themselves (**Figure 1A**). According to both physicians and PwO, the target BMI was set slightly higher for PwO with greater BMI. The target BMI was set at <24.0 kg/m^2^ by 31.5% (219/695) and 46.1% (317/688) of physicians and PwO, respectively, and 24.0–27.9 kg/m^2^ by 46.2% (321/695) and 46.4% (319/688), respectively (**Figure 1B**). Across all BMI categories, the mean (SD) target percentage weight loss was 19.6% (10.9%) according to physicians and 23.7% (10.0%) according to PwO (**Figure 1C**). The target percentage weight loss increased with BMI according to both physicians and PwO. Approximately half or more of PwO had a target percentage weight loss of at least 20% as set by physicians (48.5%; 337/695) or themselves (63.4%; 436/688) (**Figure 1D**).

Overall, the median (Q1, Q3) anticipated time to reach the weight loss target was 12 months (6, 12 months), and the median (Q1, Q3) anticipated average speed of weight loss was 1.7% (1.1%, 2.3%) of body weight per month. The anticipated time to achieve target weight and anticipated average speed of weight loss increased with the target percentage weight loss (**Figures 1E, F**). PwO and physicians desired improved patient quality of life and other physical and mental benefits for the PwO resulting from weight loss (**Figures 1G, H**).

### Actual weight loss

**Figure 2:**
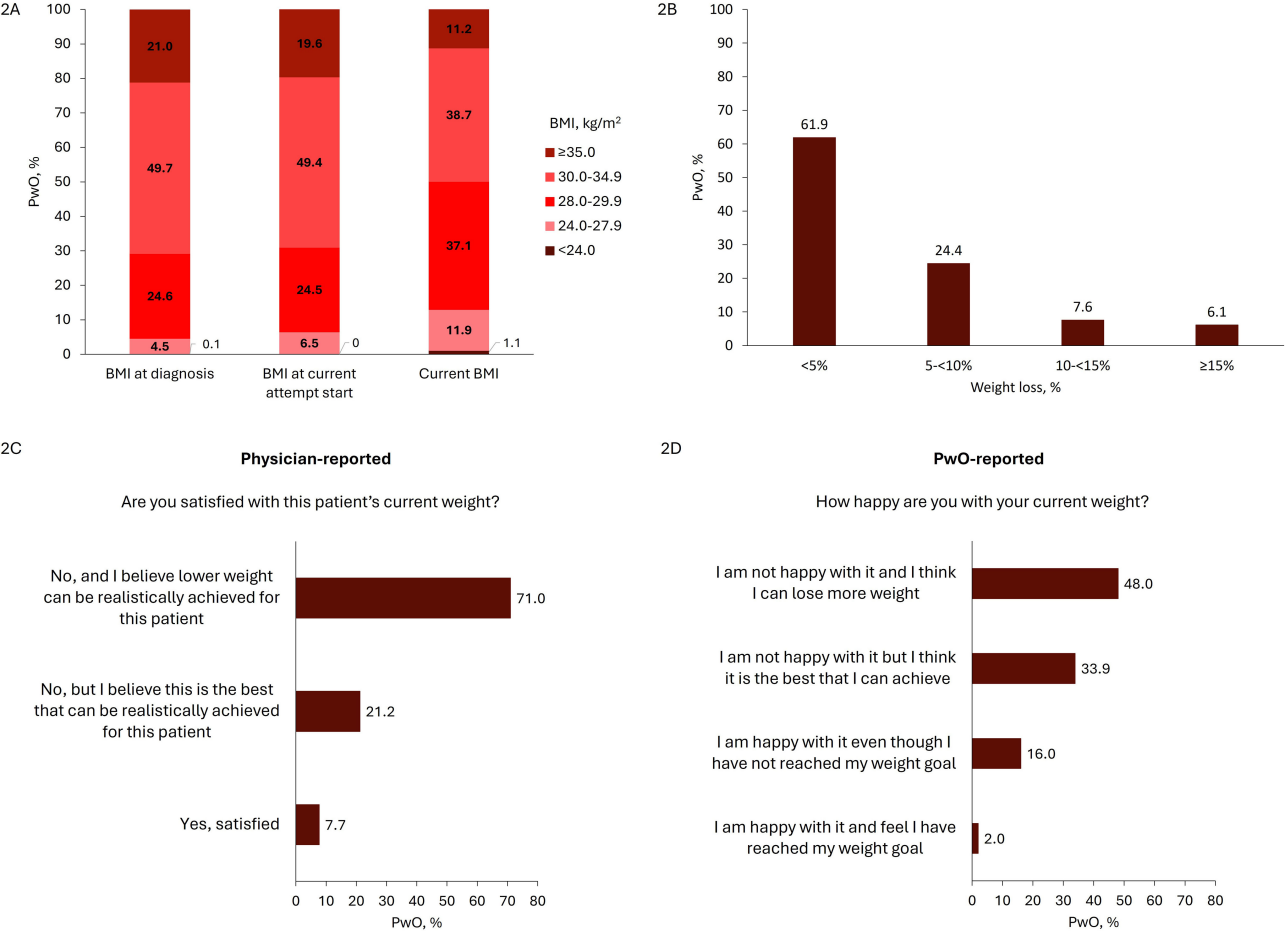
Weight loss at current attempt **(A)** BMI distribution at diagnosis, start of current attempt, and current timepoint (n=751, n=801, and n=801, respectively), **(B)** Weight loss percentage since start of current attempt (n=800), **(C)** Physician (n=801), and **(D)** PwO satisfaction with current weight loss (n=793). BMI, body mass index; PwO, people with obesity or overweight; SD, standard deviation.

Among the 751 PwO who reported weight and height at diagnosis, the mean (SD) BMI at diagnosis was 32.5 (4.5) kg/m^2^ (**Table 3**). Among 531 patients with data, the mean (SD) waist circumference was 95.0 (11.9) cm in females and 104.0 (13.5) cm in males (**Table 3**). More than two thirds of PwO (70.7%; 531/751) had a BMI ≥30.0 kg/m^2^ at diagnosis (**Figure 2A**). During the weight loss journey of PwO, the mean (SD) BMI at their heaviest was 33.5 (4.9) kg/m^2^ (**Table 3**). Most PwO had a history of multiple weight loss attempts (mean [SD] was 6.0 [4.1] per person); however, only a mean (SD) of 2.1 (1.8) attempts were under professional guidance. Most PwO (90.4%; 719/795) attempted weight loss intermittently. The main reasons for PwO ceasing to attempt weight loss were finding their diet and exercise program too hard to continue (69.3%; 551/795) and feeling that they were not losing enough weight (57.9%; 460/795) (**Table 3**).

**Table 3:** PwO weight loss journey.

	N^†^	Value
Past attempts		
BMI at heaviest, kg/m^2^, mean (SD)	636	33.5 (4.9)
BMI at diagnosis, kg/m^2^, mean (SD)	751	32.5 (4.5)
WC (female) at diagnosis, cm, mean (SD)	296	95.0 (11.9)
WC (male) at diagnosis, cm, mean (SD)	217	104.0 (13.5)
Total attempts, mean (SD)^‡^	795	6.0 (4.1)
Under the guidance of a doctor, nurse or therapist		2.1 (1.8)
Under the guidance of a diet support group		1.3 (1.1)
Support by friend(s)		1.9 (1.7)
Alone		3.1 (2.8)
Pattern of weight loss journey, n (%)^‡^	795	
On and off		719 (90.4)
Trying to lose weight all the time		76 (9.6)
Reason for stopping weight loss (multiple choice), n (%)^‡^	795	
When I find my diet and exercise program too hard to keep going		551 (69.3)
I give up when I am not losing enough weight		460 (57.9)
When I have lost enough weight		219 (27.3)
When I get side effects from the medicine I take to help me lose weight		163 (20.3)
Current attempt		
BMI at start of current attempt, kg/m^2^, mean (SD)	801	32.3 (4.5)
BMI at present, kg/m^2^, mean (SD)	801	30.9 (4.3)
Duration of current attempt, months, median (Q1, Q3)	772	6.4 (3.9, 12.1)
% weight loss since start of current attempt, mean (SD)	801	4.1 (7.4)
Achievement of BMI target set by physician at current attempt, n (%)	695	69 (9.9)
Achievement of BMI target set by the patient themselves at current attempt, n (%)^‡^	688	14 (2.0)
Weight loss speed (% weight loss/duration of current attempt [months]), median (Q1, Q3)^§^		
PwO who achieved weight loss target set by physician	58	0.69 (0.43, 1.08)^¶^
PwO who did not achieve weight loss target set by physician	552	0.43 (0.00, 0.94)
Description of weight loss journey to date, n (%)	414	
Never lost weight		94 (22.7)
Slow weight loss but regained weight since		95 (22.9)
Slow weight loss with weight loss maintenance to present		138 (33.3)
Rapid weight loss but regained weight since		56 (13.5)
Rapid weight loss with weight loss maintenance to present		31 (7.5)

^†^The denominator depends on the actual number of PwO for which each question was answered.

^‡^Data from patient-reported questionnaires (n=795); other data are from the physician-reported questionnaires.

^§^Excluding PwO with weight management duration of less than 1 month, since very short-term weight management may result in a large variation in weight loss speed.

^¶^p<0.0001 versus PwO who did not achieve weight loss target set by HCP.

BMI, body mass index; PwO, people with obesity or overweight; SD, standard deviation; WC, waist circumference.

Mean (SD) BMI was 32.3 (4.5) kg/m^2^ at the start of the current weight loss attempt (**Table 3**). The actual weight loss during the current attempt was modest; over a median (Q1, Q3) of 6.4 (3.9, 12.1) months, the mean (SD) percentage weight loss was 4.1% (7.4%) (**Table 3**). Most patients (61.9%; 495/800) only achieved <5% weight loss from the start of the current attempt (**Figure 2B**). Few PwO achieved the BMI target set by their physician (9.9%; 69/695) or by themselves (2.0%; 14/696) (**Table 3**). Median (Q1, Q3) weight loss speed (% weight loss/duration of current attempt [months]) was greater in PwO who did versus did not achieve the weight loss target set by their physician (0.69 (0.43, 1.08) vs. 0.43 (0.00, 0.94) %/month) (**Table 3**). No other meaningful differences were found between PwO who did versus did not achieve the weight loss target set by their physician (data not shown). More than half of PwO (59.2%; 245/414) had never lost weight or had regained any lost weight during the current attempt according to their physicians (**Table 3**). Most physicians and PwO were dissatisfied with the course of weight loss during the current weight loss attempt (92.3% [739/801] and 82.0% [650/793], respectively) (**Figures 2C, D**). Approximately half of PwO (47.9%; 381/795) and in almost three-quarters of PwO, physicians (71.0%; 569/801) indicated that they thought more weight could have been lost.

## Discussion

Findings from this study identified two gaps in obesity management in China. Firstly, while most physicians (89.0%) recognized the importance of treating obesity, the management of obesity may not have been prioritized, as suggested by the severity of obesity at diagnosis and the main incentive for initiating weight loss being the pre-existence of ORCs rather than increased weight alone. These observations suggest that there may be a gap between physicians’ knowledge and their actions in clinical practice regarding the treatment of obesity and ORCs. Secondly, most physicians and PwO set near-normal weight targets, with PwO desiring achievement of an even lower weight than the weight goal set by physicians. However, few PwO achieved their weight targets. Neither PwO nor physicians were satisfied with the outcomes of the current weight loss attempts, suggesting a potential unmet need for effective and acceptable methods of weight management. These findings may provide valuable insights into current weight loss management in China.

Most physicians (71.0%) recalled an increase in the number of PwO seeking health care over the past 3 years, which may reflect progress in the development of guidelines for the clinical management of obesity and introduction of obesity-related policies and programs over recent years (6, 12), as well as the increasing prevalence of obesity (1). Nonetheless, in this study, the mean BMI at diagnosis for PwO was 32.5 kg/m^2^, mean waist circumference was 95.0 cm in females and 104.0 cm in males, and most PwO were already being treated for ORCs prior to obesity diagnosis, highlighting a further need for earlier diagnosis of obesity. In China, both healthcare providers and PwO tend to have low awareness that weight management for obesity should be implemented at disease onset rather than waiting for the development of ORCs (4, 6). Nonetheless, obesity has been recognized as a disease since 2010 and was described as a chronic relapsing disease process by the World Obesity Federation in 2017 (17). Notably, most physicians (81.0%) still believed that obesity was caused by poor lifestyle choices, which may partly explain why they prioritize the treatment of ORCs instead of obesity. Barriers to the awareness of obesity control in China are complex and may include the following factors. Firstly, in traditional Chinese culture, excess bodyweight tends to be viewed positively (8, 9), which may impede public awareness of obesity as a disease and the implementation of weight loss programs. Secondly, body weight misperception is common in Chinese PwO (10, 11), with nearly half believing that they have a healthy weight (11). Moreover, even people who are aware of their increased weight may believe that they have metabolically healthy obesity and do not need to manage their weight. However, if not properly managed, metabolically healthy obesity can transition quickly to a metabolically unhealthy state, which is associated with an increased risk of developing metabolic and cardiovascular diseases (18, 19). Considering the risk of all-cause mortality and increased disability with obesity (and underweight) (20), to optimize long-term outcomes, there is a clear need to initiate weight management at the onset of obesity and before the occurrence of comorbidities (6). Therefore, further efforts are required to educate healthcare providers and PwO about obesity (12).

The weight loss goals set by physicians and PwO suggested that both recognize the importance of weight normalization, with PwO themselves often setting an even lower weight target than physicians. Although modest weight loss (5-10%) is clinically meaningful in PwO, greater weight loss (≥10%) has a more beneficial impact on ORCs (21). Nevertheless, considering long-term outcomes, BMI normalization is the optimal weight management target, which is associated with the lowest all-cause mortality (20). However, as failure to achieve an ambitious weight loss target may demoralize PwO and health-care providers, Chinese guidelines published before 2024 compromised by recommending a current clinical target of 5-15% weight loss in the first 6 months (6), even though greater weight loss may be more beneficial in the long term (21). The current Chinese guidelines recommend a staged weight management approach (22). In the initial weight loss stage, multiple weight loss milestones can be set and tailored based on the overall health status of PwO; for example, in the first 3–6 months, 10–15% weight loss for younger PwO with fewer complications, and 5–10% weight loss for older PwO with more complications. Once this has been achieved, the next goal is to set and gradually achieve an individualized optimal weight, which for most people is defined as BMI normalization. In the weight maintenance stage, the aim is to sustain the optimal weight and minimize weight cycling. Additionally, a recent meta-analysis concluded that PwO are more likely to lose weight if they receive peer support, which can be provided face-to-face (peer-led workshops and support groups), online (video training, teleconferences and social media groups) or as a combination of both approaches (23). There remains a need to develop more effective weight loss programs, which will be covered in a separate analysis of the data from this DSP.

In this study, few PwO achieved their weight loss targets and neither PwO nor their physicians were satisfied with the outcomes of the weight loss attempts. Several factors may contribute to the failure to achieve weight loss goals. Firstly, as discussed above, there may be an initial delay in obesity diagnosis. This highlights the importance of promoting early diagnosis based on the latest clinical guidelines, using factors including body shape, BMI, waist circumference, body fat mass, and visceral fat mass (22). Secondly, obesity is a chronic relapsing disease (17), yet public recognition of this is low. Our findings showed that many PwO were using methods of weight loss involving multiple intermittent attempts, most of which were not conducted under professional guidance. Thirdly, anti-obesity medications are limited in China; during the time window of the current study, the only medication approved for weight management was orlistat, which has limited weight loss efficacy (24). Furthermore, in China, anti-obesity medications are used conservatively. Chinese guidelines recommend lifestyle interventions as first-line therapy for obesity, reserving anti-obesity medications for PwO who fail to lose 5% of their body weight after 3 months of lifestyle interventions (6). However, the present results suggest that PwO tend to discontinue weight management if the lifestyle change is too difficult to follow or if they feel that they are not losing enough weight. Therefore, public education on correct weight management methods, patient support groups for weight management, and development of effective and acceptable methods of weight management are needed.

This study had some limitations. The PwO included in the survey were undergoing clinical management for weight loss, which suggests that they might have more health knowledge and willingness to control their weight compared with the general Chinese population with obesity. Moreover, data that were collected according to physicians’ recall or self-reported by PwO might be subject to information bias and should be interpreted with caution. However, physicians were randomly selected from publicly available lists of Chinese healthcare physicians to ensure the representativeness of the data. Furthermore, patient-reported questionnaires were completed in the clinical setting, which should largely ensure data quality. All information reported in the physician-reported questionnaires was derived from medical records, thereby minimizing information bias.

In conclusion, our findings revealed the potential for two main gaps in weight management in China. Firstly, the results suggest that obesity diagnosis may be delayed and that the focus is on treating ORCs rather than prioritizing the management of obesity. Secondly, while physicians and PwO appeared to recognize the need for weight control to a near-normal level, few PwO achieved their weight loss targets. This suggests the importance of improving weight management awareness among both physicians and PwO, as well as a potential unmet need for early and effective weight loss interventions.

## Data Availability

All data relevant to the analysis are included in the article. All data that support the findings of this survey are the intellectual property of Adelphi Real World. The datasets generated during and/or analyzed during the current study are available upon reasonable request to Victoria Higgins at Victoria. Higgins@adelphigroup.com.
